# Effects of exenatide on postprandial vascular endothelial dysfunction in type 2 diabetes mellitus

**DOI:** 10.1186/s12933-015-0188-1

**Published:** 2015-02-18

**Authors:** Keiichi Torimoto, Yosuke Okada, Hiroko Mori, Takashi Otsuka, Mayuko Kawaguchi, Megumi Matsuda, Fumi Kuno, Kei Sugai, Satomi Sonoda, Maiko Hajime, Kenichi Tanaka, Tadashi Arao, Yoshiya Tanaka

**Affiliations:** First Department of Internal Medicine, School of Medicine, University of Occupational and Environmental Health, 1-1 Iseigaoka, Yahatanishi-ku, Kitakyushu-shi, 807-8555 Japan

**Keywords:** Exenatide, Reactive hyperemia index (RHI), T2DM, Endothelium function

## Abstract

**Background:**

Basic studies have shown that glucagon-like peptide-1 (GLP-1) analogs exert a direct protective effect on the vascular endothelium in addition to their indirect effects on postprandial glucose and lipid metabolism. GLP-1 analogs are also reported to inhibit postprandial vascular endothelial dysfunction. This study examined whether the GLP-1 analog exenatide inhibits postprandial vascular endothelial dysfunction in patients with type 2 diabetes mellitus (T2DM).

**Methods:**

Seventeen patients with T2DM underwent a meal tolerance test to examine changes in postprandial vascular endothelial function and in glucose and lipid metabolism, both without exenatide (baseline) and after a single subcutaneous injection of 10 μg exenatide. Vascular endothelial function was determined using reactive hyperemia index (RHI) measured by peripheral arterial tonometry before and 120 min after the meal loading test. The primary endpoint was the difference in changes in postprandial vascular endothelial function between the baseline and exenatide tests.

**Results:**

The natural logarithmically-scaled RHI (L_RHI) was significantly lower after the baseline meal test but not in the exenatide test. The use of exenatide resulted in a significant decrease in triglycerides (TG) area under the curve and coefficient of variation (CV). The change in L_RHI correlated with changes in CV of triglycerides and HDL-cholesterol. Multivariate analysis identified changes in triglyceride CV as the only determinant of changes in L_RHI, contributing to 41% of the observed change.

**Conclusions:**

Exenatide inhibited postprandial vascular endothelial dysfunction after the meal loading test, suggesting that exenatide has a multiphasic anti-atherogenic action involving not only glucose but also lipid metabolism.

**Trial registration:**

ClinicalTrials.gov: UMIN000015699.

## Background

Patients with type 2 diabetes mellitus (T2DM) are at high risk for development of life-threatening atherosclerotic disease compared with healthy persons. For example, the risk of coronary artery disease is 2.0 times higher, and the risk of cerebral infarction is 2.3 times higher in these patients [1]. It is presumed that vascular endothelial dysfunction precedes clinically-evident diabetic macrovasculopathy [2] and that the former is due to various factors such as abnormal glucose and lipid metabolism, inflammation, hypertension, obesity, sedentary life style, smoking, high salt intake, and menopause [3]. Among these factors, it seems that postprandial changes in glucose and lipid metabolism are particularly important risk factors for vascular endothelial dysfunction [4].

Both clinical and experimental evidences suggest that oxidative stress and hypercytokinemia are associated with postprandial hyperglycemia [5] and that postprandial hyperlipidemia enhances the progression of atherosclerosis in patients with T2DM [6]. Therefore, correction of postprandial metabolic disorders and inhibition of vasculopathy through the above pathways could potentially prevent the progression of atherosclerosis.

Previous studies reported that incretin analogs do not only indirectly correct postprandial glucose and lipid metabolism [7], but they also inhibit vascular endothelial dysfunction by their direct protective effects on the vascular endothelium, such as enhancement of nitric oxide (NO) production [8] and anti-inflammatory action [9]. In actual clinical settings, long-term administration of sitagliptin is reported to improve vascular endothelial function [10] and exert anti-arteriosclerosis action [11]. Clinically, glucagon-like peptide-1 (GLP-1) receptor agonists are known to improve pro-atherosclerosis factors, such as glucose metabolism, lipid metabolism, blood pressure, and body weight and that their long-term effects include improvement of vascular endothelial function. In addition, one study reported that continuous intravenous infusion of GLP-1 resulted in short-term, blood glucose-independent improvement of vascular endothelial dysfunction [12]. To our knowledge, only a few studies have verified the effects of GLP-1 receptor agonists on postprandial vascular endothelial dysfunction in daily clinical practice, and only little is known at present on the mechanism of the inhibitory effects of these drugs on vascular endothelial dysfunction. The present study examined the effects of exenatide, a GLP-1 receptor agonist, on postprandial vascular endothelial dysfunction after meal loading test in Japanese patients with T2DM.

## Methods

### Study subjects

This study included 17 patients with T2DM who were admitted to the University of Occupational and Environmental Health Hospital (UOEH) or Wakamatsu Hospital of UOEH between June 2011 and February 2014 and who met the following inclusion criteria: 1) age 20 to less than 80 years; 2) no change in treatment with oral glucose-lowering agents, lipid-lowering agents, and anti-hypertensive agents during the 12 weeks preceding enrollment; and 3) ongoing treatment by diet regimen alone or by therapy with sulfonylurea, sulfonylurea plus biguanide, or sulfonylurea plus thiazolidine derivatives. Patients who met any of the following criteria were excluded from the study: 1) treatment of diabetes with insulin; 2) experience of episodes of diabetic ketoacidosis, nonketotic hyperosmolar coma, infection, or acute coronary syndrome; 3) pregnancy or possible pregnancy; 4) history of stroke or ischemic heart disease within the preceding 6 months; 5) history of pancreatitis; and 6) cardiac arrhythmia.

This study was approved by the ethics committee of the UOEH, and the subjects received written information about the study and gave consent to participate in the study.

### Study design

All 17 patients with T2DM were admitted to the hospital and underwent meal tolerance test to examine changes in postprandial endothelial function and changes in glucose and lipid metabolism (day 1). The test was repeated the next day 15 min after subcutaneous injection of 10 μg exenatide (Byetta®; AstraZeneca K.K., Osaka, Japan) (day 2). Both tests were carried out early in the morning after 12 h of fasting. With regard to the meal tolerance test, a test meal (total 450 kcal; 51.4% carbohydrate, 33.3% fat, and 15.3% protein, a recipe proposed by a working group of the Japan Diabetes Society) [13] was used, and blood was analyzed before and 30, 60, 120, and 240 min after meal loading to evaluate changes in glucose and lipid metabolism. In addition, vascular endothelial function was evaluated before and 120 min after meal loading, using a peripheral arterial tonometry (PAT) device (EndoPAT2000; Itamar Medical, Caesarea, Israel). The evaluation items related to glucose metabolism included plasma glucose and plasma immunoreactive insulin (IRI). The evaluation items related to lipid metabolism were triglycerides, high-density lipoprotein cholesterol (HDL-C), and low-density lipoprotein cholesterol (LDL-C). The area under the curve (AUC) and the coefficient of variation (CV) were calculated for plasma glucose and IRI based on data obtained at 5 measurement points from 0 to 240 min. The primary endpoint was changes in vascular endothelial function at 0 and 120 min after meal loading test conducted after exenatide, relative to the control (test without exenatide).

### Noninvasive vascular function test

The PAT-based method used for digital assessment of vascular function has been described in detail previously [14]. After an acclimatization period 30 min in a room controlled for temperature and light in the fasting state, the baseline pulse amplitude was recorded during a period of 5 min before the induction of ischemia. The latter was induced by placing the sphygmomanometer cuff on the upper arm, while the opposite arm served as a control. The PAT probes were placed on one finger of each hand. After 5 min, the blood pressure cuff was inflated to 60 mmHg above the systolic pressure or 200 mmHg for 5 min and then deflated to induce reactive hyperemia. As a measure of reactive hyperemia, the reactive hyperemia index (RHI) was calculated as the ratio of the average amplitude of the PAT signal over 1 min beginning 1.5 min after cuff deflation (control arm, A; occluded arm, C) divided by the average amplitude of the PAT signal over the 2.5-min time period before cuff inflation (baseline) (control arm, B; occluded arm, D). Thus, RHI = (C/D)/(A/B) × baseline correction. Because RHI has a heteroscedastic error structure, we used natural logarithm transformation in all analyses.

### Measurement of blood HbA1c, plasma glucose, IRI and serum lipids

Blood samples were collected early in the morning after at least 12-h fasting, through a venous line placed in the median vein using an indwelling catheter. Plasma lipid was measured with a Hitachi 7350 autoanalyzer (Hitachi Co., Tokyo, Japan). HDL-C, and triglycerides levels were determined by using an enzymatic method, and both enzymatic method and direct technique were used for LDL-C. The insulin resistance index (homeostasis model assessment of insulin resistance) was calculated according to the formula: fasting IRI (mU/L) × fasting glucose (mg/dL)/405. Hemoglobin Alc (HbA1c) was measured by high-pressure liquid chromatography using the Tosoh HLC-723 G8 (Tosoh Co., Kyoto, Japan). The HbAlc level was obtained as a national glycohemoglobin standardization program value by adding 0.4% to the value expressed as the conventional Japanese standard substance value [15].

### Statistical analysis

Values are expressed as mean ± standard error. Hypoglycemia, requiring glucose intake, developed in two patients during the meal tolerance test after exenatide administration. Excluding these two patients, the analysis included 15 patients. Wilcoxon’s signed rank test was used to compare natural logarithmic-scaled RHI (L_RHI) values at 0 or 120 min after the baseline and exenatide meal loading tests. The Friedman test was used to compare various parameters at 0, 30, 60, 120, and 240 min after meal loading. Spearman’s correlation method was used for analysis of the correlation between L_RHI and changes in glucose metabolism or lipid metabolism. Multivariate analysis used the difference between changes in L_RHI in the baseline and exenatide meal tolerance tests as the dependent variable. The independent variables were age, sex, body mass index (BMI), disease duration, and differences between changes in blood glucose AUC, changes in triglycerides CV, and changes in HDL-C CV without exenatide compared with exenatide administration. The step-up procedure was used for this analysis. Statistical analyses were conducted using SPSS Statistical Software version 19.0 (SPSS Inc., Chicago, IL), and the results were regarded as significant when the *p* value was <0.05.

## Results

### Clinical characteristics

Table 1 shows the patient characteristics. Since the administration of exenatide resulted in hypoglycemia in two patients during the meal tolerance test, they were excluded from the study. Thus, the study subjects were 15 patients with T2DM. The subjects were 15 patients (13 men and 2 women) with a mean age of 53.2 ± 2.6 (range, 35–71) years. They were mildly obese, with a mean BMI of 27.1 ± 1.5 kg/m^2^. The mean duration of diabetes mellitus was 7.0 ± 1.0 (range, 1–14) years. The mean fasting plasma glucose was 153.0 ± 9.5 (range, 114–270) mg/dL, HbA1c was 9.5 ± 0.4% (range, 7.6–13.7%), and the insulin level was 7.4 ± 1.1 (range, 2.2–16.2) μU/mL. LDL-C was 119.5 ± 9.3 (range, 64–202) mg/dL, HDL-C was 45.7 ± 3.5 (range, 29–86) mg/dL, and triglycerides was 153.0 ± 9.5 (range, 105–286) mg/dL.

**Table 1:** **Baseline characteristics**

Age (years)	53.2 ± 2.6
Gender (male/female)	13/2
Body mass index (kg/m^2^)	27.1 ± 1.5
Duration of diabetes (years)	7.0 ± 1.0
Diabetes complication	
Neuropathy	9 (60.0)
Retinopathy	2 (13.3)
Nephropathy	0 (0.0)
Diabetes therapy	
Diet only	2 (13.3)
Sulfonylurea	12 (80.0)
Pioglitazone	0 (0.0)
Metformin	3 (20.0)
Other treatments	
Lipid-lowering drugs	4 (26.7)
Antihypertensive drugs	5 (48.8)
Current smokers	9 (60.0)
Cardiovascular disease	2 (13.3)
Systolic blood pressure (mmHg)	127.4 ± 4.3
Diastolic blood pressure (mmHg)	80.6 ± 3.3
LDL-C (mg/dL)	119.5 ± 9.3
HDL-C (mg/dL)	45.7 ± 3.5
Triglycerides (mg/dL)	153.0 ± 9.5
HbA1c (%)	9.5 ± 0.4
Fasting plasma glucose (mg/dL)	153.0 ± 9.5
Immunoreactive insulin (μU/mL)	7.4 ± 1.1
HOMA-IR	2.8 ± 0.5
HOMA-β	32.2 ± 5.7
C-peptide in urine (μg/day)	101.2 ± 18.8
L_RHI	0.54 ± 0.04

Data are mean ± SE, n, or n (%). n = 15.

*Abbreviations*: *LDL-C* low-density lipoprotein cholesterol, *HDL-C* high-density lipoprotein cholesterol, *TG* triglycerides, *HbA1c* hemoglobin A1c, *HOMA-IR* homeostasis model assessment as an index of insulin resistance, *HOMA-β* homeostasis model assessment beta cell function, *L_RHI* the natural logarithmic scaled reactive hyperemia index.

Thirteen of the 15 patients were on oral hypoglycemic drugs: 1 on metformin monotherapy, 2 on metformin plus sulfonylurea, and 10 on sulfonylurea monotherapy. Two patients (13.3%) had history of cardiovascular diseases, and 4 (26.7%) were on lipid-lowering agents.

The L_RHI was 0.54 ± 0.04 (range, 0.23-1.03), and there was no significant sex-related difference in L_RHI.

### Postprandial changes in glucose metabolism and lipid profile after meal tolerance test

**Figure 1:**
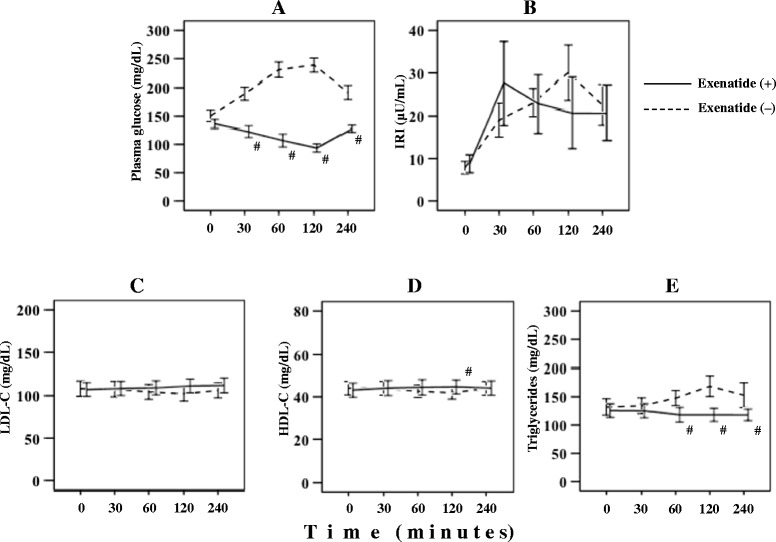
**Serial change in plasma glucose (A), immunoreactive insulin (IRI) (B), low-density lipoprotein cholesterol (LDL-C) (C), high-density lipoprotein cholesterol (HDL-C) (D) and triglycerides (E) after meal loading tests, preceded by placebo or exenatide injection.** ^#^p < 0.05 vs. without exenatide.

Glucose metabolism dynamics and lipid metabolism profile are shown in Figure 1 and Table 2. The baseline meal tolerance test was followed by significant increases in plasma glucose, IRI, and triglycerides (p < 0.001, each), with peak values registered at 2 h (Figure 1). Furthermore, the test was followed by significant decreases in LDL-C and HDL-C (p < 0.001, each), with a trough occurring at 2 h after meal.

**Table 2:** **Change in glucose and lipid metabolism after meal loading tests, preceded by placebo (baseline) or exenatide injection**

	**Baseline**	**Exenatide**	***P*** **value**
Glucose metabolism variables			
AUC of plasma glucose [(mg/dL) · hr]	855.4 ± 45.3	442.3 ± 26.4	0.001
CV of plasma glucose	0.19 ± 0.02	0.20 ± 0.01	0.776
AUC of IRI [(μIU/mL) · hr]	96.7 ± 18.5	84.6 ± 28.9	0.211
CV of IRI	0.49 ± 0.04	0.53 ± 0.05	0.427
Lipid metabolism variables			
AUC of TG [(mg/dL) · hr]	616.8 ± 68.7	475.5 ± 45.7	0.001
CV of TG	0.14 ± 0.02	0.06 ± 0.02	0.001
AUC of HDL-C [(mg/dL) · hr]	171.8 ± 12.2	177.2 ± 13.4	0.064
CV of HDL-C	0.03 ± 0.003	0.03 ± 0.003	0.730
AUC of LDL-C [(mg/dL) · hr]	419.0 ± 35.1	436.3 ± 32.8	0.221
CV of LDL-C	0.03 ± 0.003	0.03 ± 0.003	0.875

Data are mean ± SEM.

*Abbreviations*: *AUC* area under the curve, *CV* coefficient of variation, *IRI* immunoreactive insulin, *TG* triglycerides, *HDL-C* high-density lipoprotein cholesterol, *LDL-C* low-density lipoprotein cholesterol.

On the other hand, the exenatide meal tolerance test was followed by a significant decrease in plasma glucose, with a trough at 2 h after the test (p < 0.001). Furthermore, no postprandial increase was noted in triglycerides, and the triglycerides values were significantly lower at 60, 120, and 240 min after the meal compared with the corresponding values after the baseline test (p < 0.001, each).

Table 2 compares the results of the two tests. Although plasma glucose AUC was significantly lower after the exenatide test than baseline meal load test (855 vs. 442 mg/dL⋅h, p = 0.001), the IRI AUC was not different (97 vs. 85 μU/mL⋅h, p = 0.211). The exenatide meal test resulted in significant improvement in triglycerides AUC (617 vs. 476 mg/dL⋅h, p = 0.001) and triglycerides CV (0.15 vs. 0.06, p = 0.001).

### Postprandial changes in endothelial function after meal tolerance test

**Figure 2:**
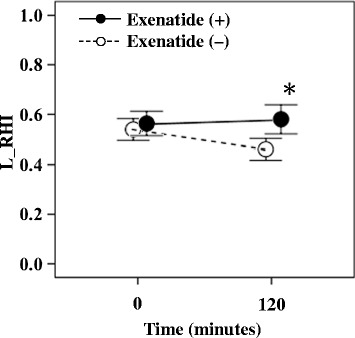
**Serial changes in endothelial function after meal loading tests, preceded by placebo or exenatide injection.** L_RHI, natural logarithmic scaled reactive hyperemia index ^*^p < 0.05 vs. baseline.

Figure 2 shows changes in L_RHI after the test meal. L_RHI was significantly lower after the baseline test meal, compared with the value before the test (0.46 vs 0.54, p = 0.029). In comparison, exenatide prevented the fall in L_RHI after the meal test (0.58 vs 0.56, p = 0.699). In addition, there was no change in pre-meal RHI without exenatide or with a single exenatide injection of 10 μg (0.54 vs. 0.56; P = 0.498). However, post-meal RHI increased significantly after a single injection of 10 μg exenatide, compared with no exenatide injection (0.46 vs. 0.58; P = 0.020). Further analysis showed that the changes in L_RHI after the baseline meal loading test did not correlate with plasma glucose AUC (r = −0.475, p = 0.074) or IRI AUC (r = 0.093, p = 0.742), but correlated with triglycerides CV (r = −0.780, p = 0.001).

Table 3 shows the relationship between changes in L_RHI on the exenatide meal tolerance test and changes in glucose and lipid metabolism. First, changes in L_RHI correlated with changes in triglycerides CV (r = −0.727, p = 0.003) and changes in HDL-C CV. Second, multivariate analysis was performed using changes in L_RHI on the baseline and exenatide meal tolerance tests as the dependent variable, and age, sex, BMI, disease duration, and changes in blood glucose AUC, triglycerides CV, and HDL-C CV on the same tests as the independent variables. The results identified changes in triglycerides CV on the two tests as the most significant contributor (contribution ratio, 41%) to the changes in L_RHI (Table 4).

**Table 3:** **Correlation coefficients between dL_RHI and clinical markers of glycemia and various nonglycemic metabolic variables**

	**dAUC**_**glucose**_	**dAUC**_**IRI**_	**dAUC**_**TG**_	**dAUC**_**HDL**_	**dAUC**_**LDL**_	**dCV**_**glucose**_	**dCV**_**IRI**_	**dCVTG**_**TG**_	**dCV**_**HDL**_	**dCV**_**LDL**_
dAUC_IRI_	−0.39									
dAUC_TG_	−0.02	0.17								
dAUC_HDL_	0.40	0.33	-.0.06							
dAUC_LDL_	−0.10	0.60*	0.04	0.35						
dCV_glucose_	0.06	0.39	−0.24	−0.12	0.50					
dCV_IRI_	−0.43	0.58*	0.29	0.02	0.37	0.35				
dCV_TG_	0.39	0.09	−0.06	−0.13	0.27	0.37	0.14			
dCV_HDL_	0.44	−0.26	−0.23	−0.46	−0.09	−0.30	0.01	0.64*		
dCV_LDL_	0.35	0.35	−0.22	−0.42	−0.24	−0.24	−0.60*	0.14	0.38	
dL_RHI	−0.37	0.33	0.24	0.16	−0.17	−0.19	0.29	−0.73**	−0.61*	−0.33

Data are results of Spearman rank correlation. *P < 0.05, **P < 0.01.

*Abbreviations*: *dAUC* change in the area under the curve, *IRI* Immunoreactive insulin, *LDL* low-density lipoprotein cholesterol, *HDL* high-density lipoprotein cholesterol, *TG* triglyceride, *dCV* change in the coefficient of variation, *dL_RHI* change in the natural logarithmic scaled reactive hyperemia index.

**Table 4:** **Results of linear multivariate analysis with dL_RHI as the dependent variable**

**Variables**	**Unstandardized coefficients**		**Standardized coefficients β**	**t**	**P value**	**95% CI**
	**B**	**SE**				
Intercept	−0.257	0.150		−1.721	0.111	−0.584, 0.069
dCV_TG_	−0.502	1.729	−0.642	−2.901	0.013	−0.878, −1.249
dAUC_glucose_	-	-	−0.203	−0.847	0.415	-
Adjusted multiple R^2^	0.412					

Multivariate stepwise regression analysis with dL_RHI as the dependent variable and age, gender, BMI, duration of the disease, dAUC_glucose_, dCV_TG_, dCV_HDL_ as the independent variables.

*Abbreviations*: *dL_RHI* change in the natural logarithmic scaled reactive hyperemia index, *dAUC* change in the area under the curve, *dCV* change in the coefficient of variation, *HDL* high-density lipoprotein cholesterol, *TG* triglyceride, *SE* Standard error, *CVD* cardiovascular disease, *95% CI* 95% confidence interval.

## Discussion

The present study indicated that single-dose exenatide can inhibit postprandial endothelial dysfunction in Japanese patients with T2DM. Furthermore, a single dose of exenatide corrected abnormalities in postprandial lipid metabolism, with particular improvement in postprandial hypertriglyceridemia, which could explain the observed improvement in postprandial vascular endothelial function. In fact, previous retrospective studies reported that exenatide inhibited the onset of cardiovascular events in patients with T2DM [16] and that liraglutide, another GLP-1 analog, improved intima-media thickening of the carotid artery [17]. These results suggest anti-atherosclerotic activity for members of the GLP-1 family, in addition to their effects on vascular endothelial function.

T2DM is known to be associated with impairment of vascular endothelial function [1], which plays a major role in atherosclerogenesis. Previous studies demonstrated that even one meal can impair vascular endothelial function in patients with T2DM [4,18]. This acute vascular endothelial dysfunction is presumed to occur through increased oxidative stress and the appearance of endothelial cell adhesion factors following enhancement of protein kinase C and nuclear factor κ-light-chain-enhancer of activated B activity, consequence to postprandial hyperglycemia and/or postprandial lipemia [19]. However, the state of high oxidative stress in various diseases, including chronic glucose and lipid metabolism disorders, hypertension, and chronic kidney disease, plays an important role in increasing the level of asymmetrical dimethylarginine [20], which is considered the main compound responsible for endothelial damage. In this study, similar to the report of Koska et al. [21], vascular endothelial dysfunction elicited by the test meal improved by single-dose exenatide. Because vascular endothelial dysfunction is a manifestation of early-stage atherosclerosis [2], we suggest that the single dose of exenatide used in this study successfully inhibited postprandial endothelial dysfunction.

It has been reported that GLP-1 receptors are expressed in vascular endothelial cells [22] and it has been reported to directly increase NO production and inhibit the expressions of endothelial cell adhesion factors [23]. GLP-1 increases NO production to improve the vasodilatory response [8]. In addition, GLP-1 is reported to inhibit overexpression of hyperglycemia-induced vascular cell adhesion molecule-1 in vascular endothelial cells [9]. In actual clinical settings, DPP4 inhibitor sitagliptin has been reported to increase GLP-1 and inhibit the expressions of endothelial cell adhesion factors such as intercellular adhesion molecule-1 (ICAM-1) and E-selectin [24], indicating GLP-1 has direct and short-term vasodilatory and anti-inflammatory effects that result in improvement of vascular endothelial function. These effects could, at least in part, explain the improvement in vascular endothelial function observed after in the present study after a single dose of exenatide.

In this study, exenatide administered before the meal loading test inhibited the postprandial increase in triglycerides. Previous studies also showed that a single dose of GLP-1 inhibits the postprandial rise in triglycerides and free fatty acids in healthy persons [25] and inhibits the postprandial rise in triglycerides and apolipoprotein B48 in patients with T2DM [26]. GLP-1 is also reported to suppress chylomicron synthesis by inhibiting apolipoprotein B48 production and triglycerides absorption through direct action on the gastrointestinal tract [7], and inhibits postprandial increase in triglycerides by reducing the gastric emptying rate [27]. It is presumed that the same mechanisms are involved in the improvement of postprandial lipid abnormalities observed in the present study.

Recent large-scale clinical studies have shown that postprandial hypertriglyceridemia increases the risk of cardiovascular events independent of other risk factors of diabetes mellitus, hypertension, and lipid metabolism during fasting [28,29]. On the other hand, it has been reported that postprandial deterioration of vascular endothelial function is associated with postprandial hyperglycemia and hypertriglyceridemia [30]. Improvement in vascular endothelial function after single-dose administration of incretin analog alogliptin [31] or exenatide [21] has also been reported to be related to improvement in postprandial lipid abnormalities. In the present study, postprandial hypertriglyceridemia correlated with postprandial decrease in vascular endothelial function, and improvement in postprandial vascular endothelial function after exenatide correlated with improvement in postprandial hypertriglyceridemia. One possible mechanism for this improvement is inhibition of oxidative stress and reduced expression of endothelial cell adhesion factor through improvement in postprandial lipid abnormalities, leading to improvement in vascular endothelial dysfunction. Such improvement seems to be due to the indirect and short-term effects of exenatide.

This study had certain limitations. First, it was an open-label study covering a small sample size with possible selection bias. It has been reported that GLP-1 formulations did not improve vascular endothelial function in T2DM patients with severe obesity [32] or vascular endothelial function in fasting [33]. Therefore, the finding needs to be validated in a larger sample size in the future. Second, the direct effect of GLP-1 on inflammatory cytokines, adhesion factors, oxidative stress factors, among others, and their effects on vascular endothelial function, was not evaluated in this study. Since GLP-1 formulations are known to suppress oxidative stress and inflammation and thus suppress vascular endothelial dysfunction [34]. Our results showed that improvement in postprandial hypertriglyceridemia was responsible for 41% improvement in postprandial vascular endothelial function, while the remaining 59% was probably related to the direct effect of GLP-1 on vascular endothelial function. Further studies are required to examine the direct effects of GLP-1 on vascular endothelial function that are independent of improvement in glucose and lipid metabolism.

## Conclusions

Administration of a single dose of exenatide before the meal loading test inhibited postprandial vascular endothelial dysfunction in patients with T2DM, suggesting multitude of activities in the anti-atherosclerotic effects of exenatide.

## Abbreviations

GLP-1: Glucagon-like peptide-1
T2DM: Type 2 diabetes mellitus
RHI: Reactive hyperemia index
L_RHI: Natural logarithmically-scaled RHI
TG: Triglycerides
CV: Coefficient of variation
NO: Nitric oxide
PAT: Peripheral arterial tonometry
IRI: Immunoreactive insulin
HDL-C: High-density lipoprotein cholesterol
LDL-C: Low-density lipoprotein cholesterol
AUC: Area under the curve
HbA1c: Hemoglobin Alc
BMI: Body mass index
ICAM-1: Intercellular adhesion molecule-1
